# Hypertensive Disorders of Pregnancy and Future Cardiovascular Health

**DOI:** 10.3389/fcvm.2020.00059

**Published:** 2020-04-15

**Authors:** Karen Melchiorre, Basky Thilaganathan, Veronica Giorgione, Anna Ridder, Alessia Memmo, Asma Khalil

**Affiliations:** ^1^Department of Obstetrics and Gynecology, Spirito Santo Hospital of Pescara, Pescara, Italy; ^2^Fetal Medicine Unit, St George's University Hospitals NHS Foundation Trust, University of London, London, United Kingdom; ^3^Vascular Biology Research Centre, Molecular and Clinical Sciences Research Institute, St George's University of London, London, United Kingdom

**Keywords:** preeclampsia, gestational hypertension, hypertensive disorders of pregnancy, essential hypertension, cardiovascular disease, heart failure, coronary artery disease

## Abstract

Hypertensive disorders of pregnancy (HDP) occur in almost 10% of gestations. These women are known to have higher cardiovascular morbidity and mortality later in life in comparison with parous controls who had normotensive pregnancies. Several studies have demonstrated that women with preeclampsia present in a state of segmental impaired myocardial function, biventricular chamber dysfunction, adverse biventricular remodeling, and hypertrophy, a compromised hemodynamic state and indirect echocardiographic signs of localized myocardial ischemia and fibrosis. These cardiac functional and geometric changes are known to have strong predictive value for cardiovascular disease in non-pregnant subjects. A “dose effect” response seems to regulate this relationship with severe HDP, early-onset HDP, coexistence of fetal growth disorders, and recurrence of HDP resulting in poorer cardiovascular measures. The mechanism underlying the relationship between HDP in younger women and cardiovascular disease later in life is unclear but could be explained by sharing of pre-pregnancy cardiovascular risk factors or due to a direct impact of HDP on the maternal cardiovascular system conferring a state of increased susceptibility to future metabolic or hemodynamic insults. If so, the prevention of HDP itself would become all the more urgent. Shortly after delivery, women who experienced HDP express an increased risk of classic cardiovascular risk factors such as essential hypertension, renal disease, abnormal lipid profile, and diabetes with higher frequency than controls. Within one or two decades after delivery, this group of women are more likely to experience premature cardiovascular events, such as symptomatic heart failure, myocardial ischemia, and cerebral vascular disease. Although there is general agreement that women who suffered from HDP should undertake early screening for cardiovascular risk factors in order to allow for appropriate prevention, the exact timing and modality of screening has not been standardized yet. Our findings suggest that prevention should start as early as possible after delivery by making the women aware of their increased cardiovascular risk and encouraging weight control, stop smoking, healthy diet, and daily exercise which are well-established and cost-effective prevention strategies.

## Introduction

Numerous epidemiological studies have proven the relationship between hypertensive disorders in pregnancy (HDP) and increased cardiovascular risk factors and diseases (CVD) later in life (1, 2). This is not surprising as several studies already demonstrated that women with preeclampsia (PE) present in pregnancy a state of impaired myocardial contractility and relaxation, biventricular systo-diastolic chamber dysfunction, adverse biventricular remodeling and hypertrophy, an impaired hemodynamic state, and indirect echocardiographic signs of localized myocardial ischemia and fibrosis (3–5). The above described structural and functional cardiovascular changes do not completely reverse at 1 year postpartum notwithstanding the unloading conditions and are known to be highly predictive for future cardiovascular adverse outcome (3, 6). Furthermore, clinical data has shown that the cardiopulmonary complications already occur in 6% of severe PE and are associated with increased maternal mortality (7). The American Heart Association has already recognized PE as independent risk factor for CVD and introduced this complication of pregnancy in the algorithms for the evaluation of future cardiovascular risk score (8). The physiopathology for this additional CVD risk is still unknown with the existing literature hypothesizing the possibility of pregnancy-induced CVD risk vs. pre-conceptional susceptibility toward an increased risk of CVD or both. The aim of this review is to elucidate the association between HDP and future cardiovascular risk factors and CVD. HDP include gestational hypertension (GH), PE or eclampsia, chronic hypertension, and PE superimposed on top of chronic hypertension. This review will focus on HDP involving new-onset hypertension after 20 weeks' gestation, but other complications of pregnancy such as normotensive small for gestational age or fetal growth restriction as well as normotensive preterm birth, placental abruption, and stillbirth will not be included. This review will focus on the future cardiovascular health of these women, but other outcomes, such as renal failure, diabetes, or other dysmetabolic conditions will not be examined.

## Search Strategy and Selection of Articles

A literature examination was accomplished to identify all reports in the English language literature (Medline, National Library of Medicine) published after 2000 until now as previous meta-analysis showed that there were not suitable papers on this topic from 1946 to 1999 (9). The search terms used were “pre-eclampsia,” “gestational hypertension,” “hypertension during pregnancy,” “hypertensive disorders of pregnancy,” “long term outcomes,” “cardiovascular risk,” “cardiovascular health,” “cardiovascular disease,” “essential hypertension,” “peripheral artery disease,” “asymptomatic atherosclerosis,” “diastolic dysfunction,” “heart failure,” “coronary artery disease,” “cerebrovascular disease.” We excluded abstracts of oral communications and posters of congresses even if available on Medline. Furthermore, the bibliography of the selected papers were dissected to further identify pertinent studies. The combined set encompassed 230 articles which were reviewed, and a total of 87 articles were considered appropriate for this review. The relevant data were extracted from the full text of the published papers.

## Results

The research yielded 59 prospective (3, 6, 10–66) and 28 retrospective studies (67–93) which were synthetically described in Table 1. The number of women analyzed in each study ranged from 58 to 1,072,330 in prospective studies and from 71 to 1,452,926 in retrospective ones. The year of pregnancy included into the studies ranged from 1952 to 2017 in prospective studies and from 1939 to 2016 in retrospective articles. The following complications of pregnancy were included into the studies: HDP, placental infarction and abruption, preterm delivery, low birth weight offspring, small-for-gestational-age fetuses, fetal growth restriction, stillbirth, gestational diabetes mellitus, and pre-gestational diabetes mellitus. The interval between delivery and cardiovascular risk assessment or between delivery and cardiovascular event occurrence were assessed in the majority of the studies, the median of follow up ranging from at least 7 weeks post-partum to more than 30 years after delivery. The median age of the women at the assessment of CV risk or at the occurrence of CV event ranged from 25 to 71 years in the different studies. The following cardiovascular risks or events were differently assessed in the numerous included studies: essential hypertension, heart failure, coronary, cerebro-vascular and peripheral artery disease, asymptomatic atherosclerosis, dyslipidemia, metabolic syndrome, type II diabetes, and renal dysfunction.

**Table 1:** Studies included in this review.

**References**	**Demographic**	**Design of the study**	**Study population**	**Year of pregnancy**	**Complications of pregnancy**	**Post-partum follow-up time in years**	**Age of the women at the assessment in years**	**Cardiovascular outcomes assessed**
Kestenbaum et al. (10)	North America (USA)	Population-based cohort study	*N =* 31,239 cases *N =* 92902 controls	1987–1998	HPD	7.8 (mean)	36 (mean)	Cardiovascular event
Sattar et al. (11)	Europe (UK)	Prospective case control	*N =* 40 cases *N =* 40 controls	1975–1985	PE	15–25 (range)	43 (median)	Essential chronic hypertension
Ray et al. (67)	North America (Canada)	Population-based retrospective cohort study	*N =* 75380 cases *N =* 950885 controls	1990–2004	PE, HDP, PA	8.7 (median)	Age at delivery28 (mean) Age at first CV event 38.3 (mean)	Coronary heart disease Cerebro-vascular disease Peripheral artery disease
Manten et al. (12)	Europe (Netherlands)	Prospective case control	*N =* 256 cases *N =* 53 controls	NA	PE	0.82 (mean) cases 0.48 (mean) controls	31 (mean) cases 33 (mean) controls	Essential chronic hypertension Cardiovascular risk
Berends et al. (13)	Europe (Netherlands)	Prospective case control	*N =* 106 cases *N =* 106 controls	1983–2004	PE and FGR	7.1 (median)	NA	Essential chronic hypertension Asymptomatic atherosclerosis
Valensise et al. (3)	Europe (Italy)	Prospective longitudinal case-control study	*N =* 107 *N =* 1119 controls	1999–2007	PE	1	34 (median) early onset PE 32 (median) late onset PE 32 (median) controls	Essential chronic hypertension Cardiac dysfunction and remodeling
Edlow et al. (14)	North America (USA)	Prospective case-control study	*N =* 79 cases *N =* 140 controls	2006	PE	0.5–1 (range)	NA	Essential chronic hypertension
Haukkamaa et al. (15)	Europe (Finland)	Cross-sectional study	*N =* 96 cases *N =* 489 controls	NA	HDP	NA	55.1 (mean)	Essential chronic hypertension Coronary heart disease Asymptomatic atherosclerosis
Lykke et al. (16)	Europe (Denmark)	Registry-based cohort study	*N =* 782287 cases	1978–2007	HDP	14.6 (median)	NA	Essential chronic hypertension Coronary heart disease Cerebrovascular disease Heart failure Peripheral artery disease Cardiovascular risk
Magnussen et al. (17)	Europe (Norway)	Registry-based cohort study	*N =* 15065 cases	NA	HDP	16.5 (median)	NA	Essential chronic hypertension Cardiovascular risk
Nijdam et al. (68)	Europe (Netherlands)	Retrospective case control	*N =* 35 cases *N =* 150 control	2000–2007	PE	2.9 (mean)	NA	Essential chronic hypertension Coronary heart disease Cerebrovascular disease
Smith et al. (18)	North America (Canada)	Prospective cohort	*N =* 70 cases *N =* 70 control	NA	PE	1	30.5 (mean)	Cardiovascular risk
Canti et al. (19)	South America (Brazil)	Cross sectional	*N =* 40 cases *N =* 14 controls	NA	PE	15.9 (mean)	39.2 (mean)	Cardiovascular risk
Ben-Ami et al. (69)	Asia (Israel)	Retrospective case-control study	*N =* 101 cases *N =* 101 controls	NA	HDP, PA, PD, SGA, abortions	NA	43.3 (mean) cases 41.9 (mean) controls	Essential chronic hypertension Coronary heart disease Cerebrovascular disease Peripheral artery disease Cardiovascular event
Lykke et al. (70)	Denmark	Retrospective cohort study	*N =* 782287	1978–2007	PD, SGA, HDP, PA and stillbirth	14.8 (median)	41.6 (mean)	Death from cardiovascular causes
Mongraw-Chaffin et al. (20)	California (USA)	Prospective cohort study	*N =* 24 cases *N =* 242 controls	1959–1967	PE	30	56 (median)	Cardiovascular risk
Callaway et al. (21)	Australia	Prospective cohort study	*N =* 191 cases *N =* 1921 controls	1981–1983	HDP	21	46.4 (mean)	Essential chronic hypertension Cardiovascular risk
Melchiorre et al. (6)	Europe (London, UK)	Prospective longitudinal case-control study	*N =* 64 cases *N =* 78 controls	2008–2009	PE	Two time points: 1 and 2 years	31 preterm PE (median) 33 term PE (median) 33.5 (median) controls	Essential chronic hypertension Asymptomatic heart failure (stage B)
Andersgaard et al. (22)	Europe (Norway)	Registry-based cohort study	*N =* 901 cases *N =* 9073 controls	NA	PE	25.4 (median)	48.8 (median)	Asymptomatic atherosclerosis Cardiovascular risk
Bhattacharya et al. (23)	Europe (UK)	Registry-based cohort study	*N =* 10917 cases *N =* 23937 controls	1950–2012	HDP	NA	NA	Essential chronic hypertension Coronary heart disease Cerebrovascular disease Peripheral artery disease Cardiovascular risk
Borna et al. (71)	Asia (Iran)	Retrospective case-control study	*N =* 345 cases *N =* 345 controls	NA	HDP	32.2 (mean) cases 31.5 (mean) control	58.1 (mean) cases 55.9 (mean) controls	Coronary heart disease
Gastrich et al. (72)	North America (USA)	Retrospective case-control study	*N =* 302 cases *N =* 864 controls	1994–2009	PE	Within 16	NA	Coronary heart disease Cerebrovascular disease Cardiovascular event
Drost et al. (24)	Europe (Netherlands)	Prospective case-control study	*N =* 339 cases *N =* 332 controls	1991–2007	PE	10	38.9 (mean)	Essential chronic hypertension Cardiovascular risk
Fraser et al. (25)	Europe (UK)	Prospective cohort study	*N =* 2172 cases	1991.1992	HDP	18	48 (mean)	Cardiovascular risk
Ray et al. (73)	North America (Canada)	Retrospective cohort study	*N =* 75242 cases *N =* 1 055 522 controls	1992–2009	PE, HDP, PA	7.8 (median)	37.8 (mean)	Heart failure C and dysrhythmias
Skjaerven et al. (26)	Europe (Norway)	Prospective cohort study	*N =* 34824 cases	1967–2009	PE	25	NA	Coronary heart disease Cerebrovascular disease Cardiovascular risk Cardiovascular event
Smith et al. (27)	North America (Canada)	Prospective longitudinal cohort	*N =* 99 cases *N =* 118 control	2003–2009	PE	1 time 1 3 time 2	30.3 (mean) time 1 30.5 (mean) time 2	Cardiovascular risk
Spaan et al. (28)	Europe (Netherlands)	Prospective longitudinal Cohort	*N =* 683 cases	1996–2010	PE	0.66	31.4 (mean)	Cardiovascular risk
Mangos et al. (29)	Australia	Prospective case control	*N =* 66 cases *N =* 35 controls	NA	HDP	PE: 3.8 (mean) GH: 2.9 (mean) Controls: 4.3 (mean)	PE: 37 (mean) GH: 36 (mean) Controls: 38 (mean)	Essential chronic hypertension Cardiovascular risk
Drost et al. (30)	Europe (Netherlands)	Longitudinal Cohort study	*N =* 689 cases *N =* 2703 controls	1987–2007	HDP	Every 5 years	38.4 (mean) time 1 46.7 (mean) time 2 50.8 (mean) time 3 54.1 (mean) time 4	Essential chronic hypertension Cardiovascular risk
Hermes et al. (31)	Europe (Netherlands)	Prospective, case-control	*N =* 94 cases *N =* 300 controls	2005–2008	HDP	2.5	As the woman was 60 years old for the extrapolation at 10-year risk Current age (34 years old) for extrapolation at 30-year risk	Essential chronic hypertension Cardiovascular risk
Scholten et al. (32)	Europe (Netherlands)	Prospective Cohort study	*N =* 1297 cases	2004–2010	PE	0.58 (median)	32 (median)	Essential chronic hypertension Cardiovascular risk
Van Rijn et al. (33)	Europe (Netherlands)	Prospective cohort study	*N =* 243 cases *N =* 374 control	1994–2007	PE	0.75 (mean)	30.5 (mean)	Cardiovascular risk
Kurabayashi et al. (34)	Japan	Cross–sectional	*N =* 1219 cases *N =* 9237 controls	2001–2007	HDP	NA	≥ 45 at time of survey	Essential chronic hypertension
NAkimuli et al. (35)	Uganda	Prospective Cohort study	*N =* 64 cases *N =* 124 controls	2009–2011	PE, Eclampsia	0.25	27.3 (mean) cases 24 (mean) controls	Persistent hypertension 3 months after delivery
Tooher et al. (36)	Australia	Observational cohort study	*N =* 7706 cases *N =* 64113 controls	2006–2009	HDP	NA	≥ 45 years age at study	Essential chronic hypertension Cerebro-vascular disease
McDonald et al. (74)	North American (Canada)	Retrospective cohort study	*N =* 109 cases *N =* 218 controls	1986–1995	PE	20 (median)	49 (median)	Asymptomatic atherosclerosis
Watanabe et al. (75)	Asia (Japan)	Retrospective cohort study	*N =* 101 cases *N =* 1,084 controls	NA	HDP	NA	46.5 (mean)	Essential chronic hypertension
Barry et al. (76)	North American (USA)	Retrospective case-control study	*N =* 49 cases *N =* 22 controls	2014	PE	>8 months	34 (mean)	Essential chronic hypertension Peripheral artery disease
Hosaka et al. (37)	Asia (Japan)	Prospective cohort study	*N =* 28 cases *N =* 785 controls	1994–1998	HDP	7 (median)	37 (mean)	Essential chronic hypertension
Black et al. (38)	North American (California)	Prospective cohort study	*N =* 5960 cases *N =* 358 controls	2005–2010	HDP	1 (median)	28 (mean)	Essential chronic hypertension
Cain et al. (77)	North American (State of Florida)	Population-based retrospective cohort study	*N =* 847 cases *N =* 1854 controls	2004–2007	HDP, PA, PD, SGA	4.9 (median)	25 (median)	Cardiovascular event
Ray et al. (78)	North America (Canada)	Population-based retrospective cohort study	*N =* 362 cases *N =* 1623 controls	Delivery was at least 90 days preceding the index coronary artery revascularization date (1993–2012)	PE, HDP, PA	11.3 (mean) cases 14.2 (controls)	44.7 (mean) controls 46.5 (mean) controls	Death after coronary artey revascularization
White et al. (36)	North America (USA)	Prospective, cohort study	*N =* 40 cases *N =* 40 controls	1976–1982	PE	30 (median)	59.5 (mean)	Essential chronic hypertension Coronary heart disease
Behrens et al. (37)	Europe (Denmark)	Prospective cohort study	*N =* 23 235 cases *N =* 459.737 controls	1995–2012 (first cohort) 1978–2012 (second cohort)	HDP	10 (mean), first cohort 20 (mean), second cohort	30 (median)	Essential chronic hypertension
Breetveld et al. (41)	Netherlands	Prospective longitudinal cohort study,	*N =* 69 cases	2005–2007	PE	1 and 4 y post-partum	32 (mean) at 1sr assessment 35 (mean) at 2nd assessment	Asymptomatic heart failure (stage B)
Facca et al. (79)	South American (Brazil)	Retrospective cohort study	*N =* 25 cases *N =* 60 controls	1976–2016	HDP	16 (mean)	46 (mean)	Essential chronic hypertension Peripheral artery disease
Fatma et al. (72)	Asia (India)	Retrospectivecase control study	*N =* 50 cases *N =* 50 controls	NA	PE	NA	20–45 (range)	Essential chronic hypertension Peripheral artery disease
Ghossein-Doha et al. (42)	Netherlands	Cross-sectional cohort study	*N =* 107 cases *N =* 41 controls	NA	PE	4–10	36 (mean) cases 40 (mean) controls	Asymptomatic heart failure (stage B)
Grandi et al. (43)	Europe (UK)	Population-based cohort study	*N =* 5399 cases *N =* 141349 controls	1990–2013	HDP	4.7 (median)	29.5 (mean)	Essential chronic hypertension Cardiovascular event
Mito et al. (44)	Asia (Japan)	Prospective cohort study	*N =* 25 cases *N =* 746 controls	2003–2005	HDP	5 (median)	40.3 (mean)	Essential chronic hypertension
Orabona et al. (81)	Europe (Italy)	Retrospective case–control study	*N =* 109 cases *N =* 60 controls	2007–2013	PE	4 (median)	38 (median)	Cardiac dysfunction and remodeling
Tooher et al. (82)	Australia	Retrospective cohort study	*N =* 4387 cases *N =* 27262 controls	1980–1989	HDP	20 (median)	48 (median)	Essential chronic hypertension Coronary heart disease Cerebrovascular disease
Wang et al. (83)	Asia (China)	Retrospective cohort study	*N =* 94 cases *N =* 1167 controls All GDM patients	2005–2009	HDP with GDM	2.29 (mean)	33 (mean)	Essential chronic hypertension
Benschop et al. (45)	Europe (Netherlands)	Prospective cohort	*N =* 200	2011–2017	Severe PE	1 (median)	31.6 (mean)	Essential chronic hypertension
Bergen et al. (46)	Europea (Netherlands)	Prospective cohort study	*N =* 300 cases *N =* 4612 controls	NA	HDP	6 (median)	30 (mean)	Essential chronic hypertension Cardiac dysfunction and remodeling
Bokslag et al. (84)	Europe (Netherlands)	Retrospective case control study	*N =* 131 cases *N =* 56 controls	1998–2005	Early onset PE	9–16 (range)	45 (mean)	Essential chronic hypertension Asymptomatic heart failure (stage B) Peripheral artery disease
Breetveld et al. (47)	Europe (Netherlands)	Prospective case control study	*N =* 67 cases *N =* 37 controls	2009–2011	PE	5.3 (median)	36 (median)	Asymptomatic heart failure (stage B) Peripheral artery disease
Chen et al. (85)	Asia (Taiwan)	Population–based retrospective cohort study	*N =* 29.186 cases *N =* 116.744 controls	2000–2013	HDP	5.72 (mean)	NA	Heart failure
Cho et al. (86)	Asia (Korea)	Retrospective observational cohort study	*N =* 148 cases *N =* 1762 controls	2004	PE	8 (mean)	NA	Essential chronic hypertension
Clemmensen et al. (48)	Europe (Denmark)	Observational cohort study	*N =* 53 cases *N =* 40 controls	1998–2008	PE	12 (median)	41 (mean)	Coronary flow velocity reserve
Ditisheim et al. (49)	European (Switzerland)	Prospective cohort study	*N =* 115 cases *N =* 41 controls	2010–2013	PE	0.11–0.23 (range)	33.7 (mean)	Essential chronic hypertension
Dunietz et al. (87)	North American (USA)	Retrospective cohort study	*N =* 301 cases *N =* 366 controls	1998–2004	HDP	11 (mean)	38 (mean)	Essential chronic hypertension
Egeland et al. (50)	Europe (Norway)	Prospective cohort study	*N =* 1480 cases *N =* 58.547 controls	2004–2009	HDP, pre-gestational DM, GDM, PD, FGR	7.1 (mean)	NA	Essential chronic hypertension
Escouto et al. (51)	Europe (UK)	Prospective longitudinal cohort study	*N =* 412 cases *N =* 65 controls	2009–2013	HDP	0.13 (median)	29.6 (mean)	Cardiovascular risk
Fossum et al. (88)	Europe (Norway Netherlands)	Population based retrospective cohort study	*N =* 13.348 cases *N =* 164.883 controls	1967–1998	HDP	18 (mean)	41 (mean)	Essential chronic hypertension
Grandi et al. (52)	North American (Canada)	Population-basedprospective cohort study	*N =* 5399 cases *N =* 141,349 controls	1999–2015	HDP	5 (median)	29.5 (mean)	Essential chronic hypertension Cardiovascular event
Hauspurg et al. (53)	North American (USA)	Prospective cohort study	*N =* 61 cases *N =* 254 controls	NA	HDP	0.58 (mean)	23,9 (mean)	Essential chronic hypertension
Jarvie et al. (89)	North American (USA)	Retrospective cohort study	*N =* 108.875 cases *N =* 1.344.051controls	2004–2010	HDP	3 (median)	27.2 (mean)	Acute myocardial infarction Stroke Heart failure
Kuo et al. (90)	Asia (Taiwan)	Retrospective longitudinal study	*N =* 1295 cases *N =* 5180 controls	1996–2010	PE or Eclampsia	9.8 (median)	30 (median)	Essential chronic hypertension Heart failure Cerebro-vascular disease
Markovitz et al. (54)	Europe (Norway)	Prospective cohort study	*N =* 7936 cases *N =* 18608 controls	1967–2008	HDP, SGA, PD	8.2 (median)	52 (median)	Cardiovascular event
Riise et al. (55)	Europe (Norway)	Prospective cohort study	*N =* 41 434 cases *N =* 576155 controls	1980–2009	HDP, SGA, PD	14.3 (median)	NA	Coronary heart disease Cerebro-vascular disease Cardiovascular event
Soma-Pillay et al. (56)	Africa (South African)	Prospective, case control study	*N =* 96 cases *N =* 45 controls	2013–2015	PE	1 (median)	28 (mean)	Cardiac dysfunction and remodeling
Stuart et al. (57)	North American (USA)	Observational cohort study	*N =* 5386 cases *N =* 53274 controls	1989–2009	HDP	28 (median)	55 (mean)	Essential chronic hypertension
Theilen et al. (91)	North American (USA)	Retrospective cohort study	*N =* 57,384 cases *N =* 114,768 controls *N =* 4722 cases deceased *N =* 7172 controls deceased	1939–2012	HDP	cause of death for deaths occurring at age ≤50 years vs. age >50 years	NA (mean age at childbirth 26)	Coronary heart disease Cerebro-vascular disease
Timpka et al. (58)	Europe (Sweden)	Prospective cohort study	Cohort 1 *N =* 952 cases *N =* 6600 controls Cohort 2 *N =* 658 cases *N =* 4702 controls	1955–1997	HDP, LBW offspring (<2500 g)	20 (median)	50 (mean) Cohort 1 60 (mean) Cohort 2	Cardiovascular risk
Zoet et al. (59)	Europe (Netherlands)	Multicenter, prospective cohort study	*N =* 164 Cases *N =* 387 Controls	NA	HDP	10–20 (range)	48.4 (mean)	Asymptomatic atherosclerosis
Akhter et al. (60)	Europe (Sweden)	Case control study	*N =* 23 cases *N =* 35 controls	2008–2011	PE	7	39 (median)	Asymptomatic atherosclerosis
Clemmensen et al. (61)	Europe (Denmark)	Case control study	*N =* 49 cases *N =* 39 controls	1998–2008	PE	12 (median)	41.5 (mean)	Coronary heart disease
Haug et al. (62)	Europe (Norway)	Prospective cohort study	*N =* 2119 cases *N =* 21766 controls	1984–2008	HDP	18 (median)	49 (mean)	Cerebro-vascular disease Coronary heart disease
Hromadnikova et al. (63)	Europe (Czech Republic)	Prospective cohort study	*N =* 186 cases *N =* 90 controls	2007–2013	HDP, FGR	5.4 (mean)	38 (median)	Essential chronic hypertension Cardiovascular event
Groenhof et al. (64)	Europe (Netherlands)	Population-based cohort study	*N =* 1005 cases *N =* 1811 controls	1997–2012	HDP	15 (median)	48.6 (median)	Essential chronic hypertension
Orabona et al. (92)	Europe (Italy)	Retrospective Case control study	*N =* 60 cases *N =* 30 controls	2009–2013	PE	2.35 (mean)	37 (mean)	Cardiac dysfunction and remodeling
Riise et al. (65)	Europe (Norway)	Population-based prospective cohort study	*N =* 1246 cases *N =* 18829 controls	1980–2003	HDP	10.7 (mean)	37.2 (mean)	Cerebro-vascular disease Coronary heart disease Cardiovascular risk Cardiovascular event
Sia et al. (93)	North American (Canada)	Retrospective case control study	*N =* 244 cases (Coronary artery disease) *N =* 246 controls	NA	HDP	NA	NA	Coronary heart disease
Timokhina et al. (66)	Russia	Prospective observational case-control	*N =* 90 cases *N =* 55 controls	2012–2015	PE	0,17 (mean) time 1 0,5 (mean) time 2	31,7 (median)	Cardiac dysfunction and remodeling

*NA, not available; PE, pre-eclampsia; FGR, fetal growth restriction; HDP, hypertensive disorders of pregnancy; SGA, small for gestational age; LBW, low birth weigth; PD, preterm delivery; PA, placental abruption; GDM, gestational diabetes mellitus*.

The following outcomes will be analyzed in this review:

- Essential hypertension
- Peripheral artery disease
- Asymptomatic atherosclerosis
- Asymptomatic heart failure
- Heart failure
- Coronary artery disease
- Cerebrovascular disease.

## Discussion

### Essential Hypertension

All authors who addressed the issue of the relationship between HDP and future development of essential hypertension unanimously found that women who developed HDP have an increased risk of having high blood pressure later in life (Table 1). There seems to be a “dose-dependent” effect of HDP and future risk of developing chronic hypertension depending on the severity of the hypertension in pregnancy, the onset of the complication in pregnancy, the need of iatrogenic preterm delivery, the association to fetal growth disorders and the numbers of pregnancies complicated by HDP. In particular Hauspurg et al. found that “*HDP were associated with an aOR of 1.86 (95%CI 1.37–2.52) of development hypertension at one year with PE having an aOR of 2.35 (95%CI 1.63–3.41) whereas GH having an aOR of 1.61 (95%CI 1.09–2.39)”* (53). Moreover, “*women who had PE showed an aOR of 3.23 (95%CI 1.56–6.68) of hypertension with abnormal biomarkers later in life”* (53). Other authors found that early vs. late onset PE had a higher risk of developing chronic hypertension late in life (3, 6). Furthermore, Tooher et al. found that “*the severity of hypertension in pregnancy tracked with increased risk of future hypertension”* (82), while subsequent pregnancies did not seem to confound a first episode of HDP and later CVD (82). Moreover, in a recent meta-analysis. Brouwers et al. have demonstrated that subjects with recurrent PE had a higher risk of future essential hypertension than formerly PE women with a successive unaffected gestation (RR 2.3, 95% CI 1.9 to 2.9) (94). The relation between HDP, future occurrence of essential hypertension and other pre-pregnancy CV risk has also been assessed to try and isolate the effect of pregnancy on this outcome. On this subject, Cho et al. found that the development of high blood pressure in pre-eclamptic women was related to pre-pregnancy factors such as family history, obesity and high blood pressure (86). This latter result is only partially confirmed by Mito et al. who found that women who had HDP presented an higher risk of developing high blood pressure 5 years after the index pregnancy “*even after adjusting for confounding factors such as age, body mass index, family history of hypertension and salt intake (odds ratio 7.1, 95% CI, 2.0–25.6, P* < *0.003)”* (44). Bergen et al. also found that although adjustment for BMI attenuate the relationship between HDP in childbirth age and future chronic hypertension by 65%, however it remained significant (46). In contrast, Wang et al. (83) found that “*history of HDP had a four-fold multivariable-adjusted risk (95% CI 2.29–6.24) of hypertension,”* while no influence was established by pre-conceptional anthropometric indices on the incidence of high blood pressure after delivery (83). A stratification of the risk of hypertension depending on the year post-partum after a gestation affected by HDP has also been addressed in several studies. Interestingly, Behrens et al. found that the risk of hypertension associated with HDP was high closely after a complicated gestation and continued for more than two decades (40). About 30% of subjects who suffered from a HDP may develop high blood pressure within 10 years after a complicated gestation; “*this risk was 12- to 25-fold higher in the first year, up to 10-fold in 10 year, 2-fold after 20 year”* (40). In term of number of women to be screened to detect one case of essential hypertension, Groenhof et al. found that at the age of 35, 9 women with HDP needed to be screened to detect 1 clinically relevant hypertension (64). The risk of developing essential high blood pressure after a gestation affected by HDP has also been correlated to the presence of specific echocardiographic findings (6). Specifically, Melchiorre et al. found that formerly pre-eclamptic normotensive women with moderate-severe echocardiographic left ventricle (LV) anomalies identified at 1 year after delivery were more likely to develop high blood pressure at 2 years after delivery (50%) in comparison to those with normal LV function/geometry or mild LV dysfunction/remodeling (3.5%) with a relative risk of chronic high blood pressure in women with LV moderate-severe abnormalities of 14.5 (95% CI 5.14 to 40.89, *P* < 0.001) (6). In the above mentioned study the severity of LV dysfunction and hypertrophy was graded according to the European Association and American Society of Echocardiography guidelines (EAE/ASE) (95, 96). Specifically: LV remodeling-hypertrophy was defined mild, moderate or severe if LV mass/body surface area (g/m^2^) was between 96 and 108, 109 and 121 or ≥122, respectively; LV diastolic dysfunction was defined as mild in the case of impaired myocardial relaxation pattern (grade I), moderate in the case of pseudo-normal filling pattern (grade II) or severe if restrictive filling was seen (grade III) accordingly to the EAA/ASE diagnostic algorithms for the diagnosis and grading of diastolic dysfunction (96). LV systolic function was graded based on the ejection fraction value (EF) as mildly, moderately or severely abnormal if EF (%) was between 45 and 54, 30 and 44 or <30. This finding has been confirmed by the subsequent echocardiographic studies (97).

### Peripheral Artery Disease

HDP have also been associated with increased risk of developing peripheral artery disease (98).

“Peripheral artery disease” (PAD) refers to an abnormal narrowing of arteries other than those that supply the heart or brain, commonly caused by atherosclerosis. PAD most commonly affects the lower extremities vessels (99). “*Depending on the degree of narrowing at each vascular site, a range of severity of symptoms may occur, while many patients will remain asymptomatic throughout their life. Occasionally acute events occur, often associated with thrombosis and/or embolism and/or occlusion of a major artery”* (ESC task force PAD) (99).

Ray et al. in their population-based retrospective cohort study on more than one million women, assessed the association between HDP, placental abruption and infarction (defined as maternal placental syndromes) and the occurrence of hospital admission or revascularization for PAD at least 90 days after the delivery discharge date (67). They found that the risk of PAD in women who suffered from maternal placental syndromes was 3.8 (2.4–5.9) higher than that of women who had an uneventful pregnancy (67). The future risk of PAD remained significant after adjusting for traditional risk factors for CVD, including maternal smoking and metabolic syndrome (adjusted risk 3: 1.9–4.8) and was highest in women who had HDP in combination with fetal compromise, compared to women who had not (67). Lykke et al. also found that severe PE increased the risk of subsequent thromboembolism of 1.9-fold (range 1.35–2.70) and that the relationship was “dose-dependent” (16). Subsequent studies confirmed the increased risk of PAD in women who had HDP.

### Asymptomatic Atherosclerosis

Several studies have addressed the issue of the relationship between HDP and asymptomatic/pre-clinical atherosclerosis (Table 1). “Asymptomatic atherosclerosis” refers to the coronary/carotid artery inflammatory disease while the condition is still in a subclinical stage but the presence of atherosclerosis can be well-identified and quantified by several invasive and non-invasive techniques, including coronary angiography, ultrasonography, computed tomography, and magnetic resonance imaging (100). In a prospective cohort study, “*a history of PE was associated with an increased risk of coronary artery calcifications (CAC)* >*30 years after the index pregnancy”*, even after controlling individually for traditional risk factors although this association was not more significant when corrected for current hypertension (39). Specifically, the authors found that the odds of presenting a higher CAC score was 3.54 (CI: 1.39–9.02) times greater in formerly pre-eclamptic women compared to women who did not develop PE without adjustments and it was 2.61 (CI: 0.9–7.14) times greater after correction for current high blood pressure (39). On the contrary, the association between CAC score and history of PE remained significant after adjusting for body mass index alone (odds: 3.20; CI: 1.21–8.49) (39). It has been also shown that one third of subjects who had PE express features of coronary atherosclerosis on vascular computed tomography imaging as compared to one fifth of women from the reference group imaging and this result is manifest as early as at age 45–55 years when women are on average 16 ± 6 years postpartum (59). In contrast, a prospective case-control study showed that the “*average maximum carotid intima media thickness (CIMT) was similar among women with vs. without PE (0.831 mm vs. 0.817, p* = *0.38), and PE was not a significant predictor of CIMT in a multiple linear regression model (p* = *0.63), despite more electrocardiograms compatible with coronary disease”* (74). Specifically, the authors of the above mentioned prospective study defined “abnormal electrocardiograms” the ones with at least one of the following: the presence of Q waves/isolated infarct, new left bundle branch block, ST elevation, ST depression, T wave inversion (74). The conventional measurement of common carotid artery intima-media thickness (CCA-IMT) does not represent this (60) as opposed to measurement of the individual CCA intima and media thicknesses which visibly reflect augmented vascular risk (60).

### Asymptomatic Heart Failure

Several studies assessed the relationship between HDP and the subsequent development of asymptomatic (stage B) or symptomatic (stage C) heart failure (97, 101). The American Heart Association and American College of Cardiologists define asymptomatic stage B heart failure as any subject being affected by LV hypertrophy or dysfunction (102). At this regard, it should be taken into account that several echocardiographic studies in pregnancy already showed a state biventricular dysfunction and hypertrophy, low cardiac output and high total vascular resistancein pre-eclamptic women in pregnancy vs. normotensive matched pregnant controls (3–5) A subsequent prospective longitudinal follow-up study of PE vs. controls assessed at 1 and 2 years post-partum showed, integrating conventional echocardiography, color and pulsed wave tissue Doppler, strain and strain rate techniques, that the prevalence of asymptomatic stage B heart failure (HF-B) at one year postpartum follow-up was significantly higher in preterm vs. term preeclampsia and controls (70 vs. 25 vs. 10%, respectively; *p* < 0.001), but not in term preeclampsia vs. controls (6). Similarly, moderate-severe left ventricular (LV) dysfunction and remodeling (according to the EAE/ASE diagnosis and grading of abnormal LV structure and function) were significantly more prevalent in formerly preterm PE women compared with those who had term preeclampsia and controls (3, 6). Similarly, Soma-Pillay et al. showed that women with early onset PE had an increased risk of diastolic dysfunction at 1 year post-partum (RR 3.41, 95% CI: 1.11–10.5, *p* = 0.04) and regardless of the presence of chronic hypertension (56). Breetveld et al. in their prospective longitudinal cohort study confirmed that the prevalence of HF-B was consistently high (1 in 4) amongst formerly PE women at 1 and 4 years postpartum (41). Moreover, in a subsequent observational case-control study, he confirmed that the prevalence of HF-B in formerly PE women was three-fold higher than that detected for healthy parous controls (25 vs. 8%, *P* < 0.05) at more than 4 years post-partum (47). The increased risk of asymptomatic HF in formerly PE women was further confirmed by Ghossein-Doha et al. who also found that prehypertension increased this risk significantly, while metabolic syndrome elements did not (42). Orabona et al. conducted a prospective study on hemolysis, elevated liver enzymes, and a low platelet count syndrome (HELLP) and PE women and also documented in both HELLP and PE groups a higher prevalence of LV concentric remodeling, diastolic dysfunction and reduced LV ejection fraction at 6 months to 4 years post-partum vs. matched healthy controls (81). Bokslag et.al. also found that history of PE predisposes in middle age, 9–16 years after pregnancy, to worse LV diastolic function, which could increase the likelihood of later heart failure with preserved ejection fraction (84).

### Heart Failure

Several large registry-based studies addressed the issue of the relationship between HDP and symptomatic heart failure. Specifically, in a retrospective registry-cohort study on short term cardiovascular outcome in women who had HDP, Jarvie et al. scrutinized all hospital-based deliveries in Florida from 2004 to 2010 and following cardiovascular (CV), non-CV and any second hospitalization to any Florida hospital within 36 months of index delivery excluding subsequent deliveries (89). They found that “*women with HDP had twice the risk of CV readmission within 3 years of delivery (6.4 vs. 2.5/1,000 deliveries; P* < *0.001), with higher rates among African American women. Heart failure was the most common reason for CV readmission accounting for 78.6% of all CV readmissions and 84.4% of CV readmissions in women with HDP”* (89). Furthermore, in a retrospective longitudinal registry study on long term outcome in women who had HDP, Kuo et. al. using National Health Insurance Research Database, found that formerly PE and eclamptic women were at increased risks of congestive heart failure (hazard ratio 9.1, and 7.4, respectively) with a drastic increase of congestive heart failure occurrence at 3 and 10 years since the index pregnancy (90). Similarly, Chen et al. in a retrospective cohort study found that heart failure incidence was greater in the HDP group than controls (9.83 vs. 1.67 per 10,000 person-years), was more likely to develop within 5 years and that severe or recurrent HDP had a greater risk (85).

### Coronary Heart Disease

Numerous studies have assessed the relationship between HDP and subsequent coronary heart disease (Table 1). “Coronary artery disease” refers to the clinical manifestations of atherosclerosis and comprehends a range of diseases that result from atheromatous change in coronary vessels. Specifically, it includes the following: (stable and unstable) angina pectoris, myocardial infarction, sudden cardiac death (100).

**Figure 1:**
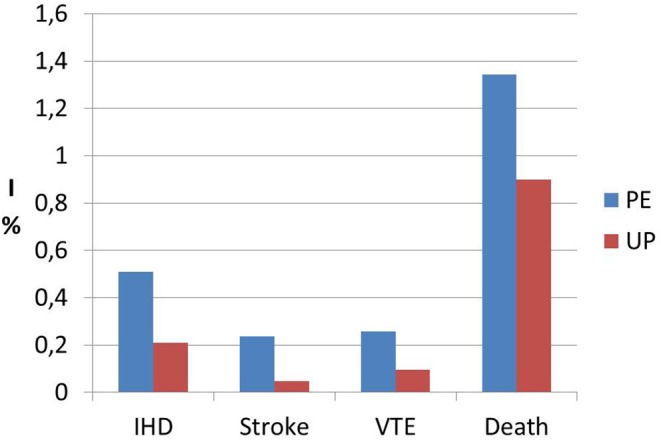
Incidence data (I) of ischemic heart disease (IHD), stroke, venous thromboembolism (VTE) and death from any cause in formerly PE women (PE) vs. women who had an uneventful pregnancy (UP). Modified from Bellamy et al. (1).

**Figure 2:**
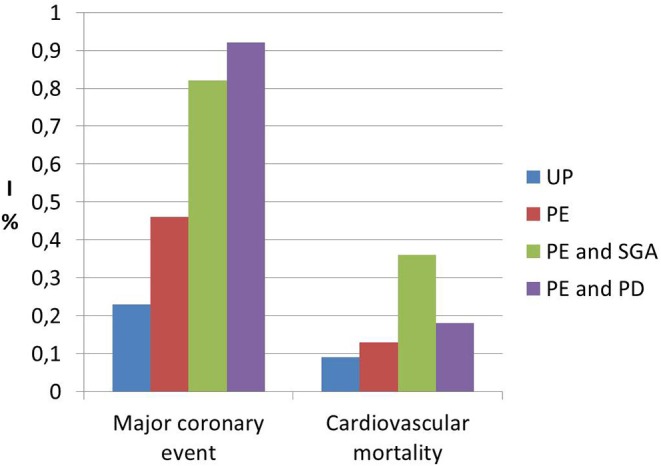
Incidence data (I) of future risk of major coronary events and cardiovascular mortality in formerly PE women (PE) vs. women who had an uneventful pregnancy (UP), showing the dose effect response of specific characteristics of pregnancy with preeclamspia such as small for gestational age (SGA) and preterm delivery (PD). Modified from Riise et al. (55).

**Figure 3:**
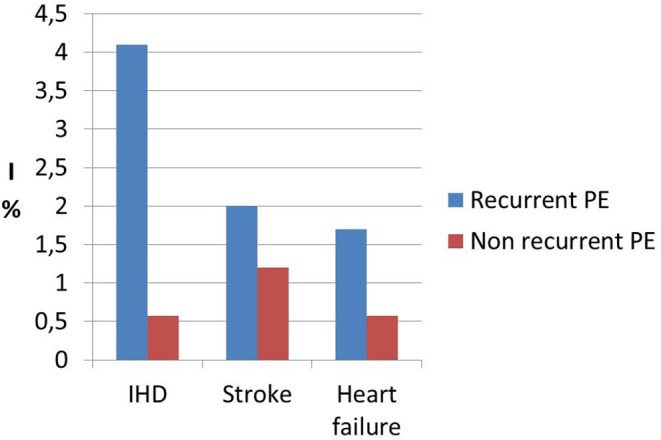
Incidence data (I) of ischemic heart disease (IHD), stroke and heart failure in recurrent preeclamspia women (recurrent PE) vs. women affected by a single pregnancy with PE and subsequent normal pregnancy (not recurrent PE) showing the “dose effect” response of recurrent preeclampsia. Modified from Brouwers et al. (94).

Interestingly, a previous study on cardiac function and structure in pre-eclamptic women showed the presence of echocardiographic findings suggestive of segmental myocardial ischemia and fibrosis (4–6). Specifically, significantly higher prevalence of basal septal post-systolic shortening, identified by Color Tissue Doppler-derived strain analysis, associated to septal bugling and segmental abnormal myocardial strain and strain rate indices were found in a minority of pre-eclamptic women with severe disease vs. normotensive pregnant controls and these echocardiographic findings have been associated in human autopsy studies and in animal *in vitro* studies to regional myocardial ischemia (4–6). Not surprising the prevalence of ischemic heart disease in formerly PE women is significantly higher than in parous controls with an uneventful pregnancy (1, 101) (Figure 1). As well as it has been demonstrated in chronic hypertension, this higher risk of developing coronary artery disease in women who had HDP show a “dose dependent effect,”, being higher in the presence of a more severe disease in pregnancy, in the case of associated fetal growth abnormalities and iatrogenic preterm birth and if PE recurred in subsequent pregnancies (55, 94) (Figures 2, 3). Moreover, the association was not explained by adjustment for confounding variables, although it was attenuated by the presence of other CV risk factors (55, 94). In particular, Tooher et. al. directed a retrospective cohort study on all subjects who delivered at a tertiary hospital in Sydney between the years 1980 and 1989 (*n* = 31,656) of whom 4,387 had HDP (82). The whole cohort were studied for linkage analysis to future CVDs and he found that the formerly HDP women were at increased risk of admission for future CVD vs. normotensive parous controls (OR 2.1; 95% CI, 1.7–2.6) (82). The median time from the index gestation to the occurrence of CVD was 20 years with a range of 3–29 years (82). Another study demonstrated that among a cohort of subjects with acute coronary syndrome, positive history of pregnancy adverse outcome was related with more severe disease and worse outcome (103). In particular, at presentation with acute coronary syndrome, women who had PE were younger and had more conventional CV risk factors such as essential high blood pressure and an increased soluble fms-like tyrosine kinase:placental growth factor ratio compared to women who had uncomplicated gestation (103). There was also an increased risk of recurrence of acute coronary syndrome at 1 year in women with previous PE (hazard ratio, 6.8) (103). Moreover, women with ≥2 complicated gestation had an increased cardiac mortality risk as shown by Theilen et al. (aHR = 3.3, 95% CI 2–5.4) (91). A large registry-study in Norway also revealed that GH was associated with increased risk of subsequent cardiovascular disease and the highest risk was noticed when GH was combined with fetal growth abnormalities infants and/or preterm delivery (65). Subsequent studies confirmed this association between HDP and increased risk of myocardial infarction at 40–70y with an HR of 2.08 for formerly PE women and 1.56 or formerly GH women (62). Furthermore, an interesting study on the relationship between coronary flow velocity reserve (CFVR) by Doppler echocardiography and previous HDP found that the mean coronary flow velocity reserve was significantly impaired in the early-onset than in the late-onset PE and in the control group with a positive relation between gestational age at PE diagnosis and coronary flow velocity reserve which notable persisted significant after adjustment for conventional cardiac risk factors such as body mass index, blood pressure, and glycated hemoglobin (61).

### Cerebro-Vascular Disease

There is general agreement on the link between HDP and subsequent risk of stroke (Table 1). It has been demonstrated that not only PE/Eclampsia increase the risk of cerebrovascular events with an HR of 10.7 (*p* < 0.0001) (90), but Haug et al. (62) and Tooher et al. (82) is also associated with increased risk of stroke. A large population-based study found that formerly GH women had a 1.3-fold (95% CI 0.9–1.7) higher risk of suffering from cerebrovascular disease compared with parous controls with uncomplicated gestation (55). In general, HDP women seem to have an increased risk of cerebrovascular events (HR 1.47, 1.15–1.87) at 40–70 years (62). Moreover, no significant difference was found in relation to the use of antihypertensive drugs or the duration of HDP and subsequent hospitalization for stroke (82). Again, there seems to be a “dose dependent” effect of HPD on cerebrovascular mortality, with women who had more than two complicated gestation showing an increased mortality from stroke (aHR = 5.10), as well as the other causes of cardiovascular mortality, compared to parous women who had only 1 or 0 pregnancies complicated by HDP (1, 91, 94) (Figures 1, 3).

## Conclusions

HDP is associated with increased risk of cardiovascular diseases later in life. This is not surprising as several studies have demonstrated that women with PE present in a state of cardiac dysfunction, ventricular hypertrophy and indirect echocardiographic signs of localized myocardial ischemia and fibrosis (103, 104). Moreover, the structural and functional cardiovascular changes do not completely reverse at 1 year post-partum and are known to have strong prognostic value for future cardiovascular morbidity and mortality in non-pregnant subjects (104). The relationship is stronger in the case of severe or early-onset HDP, concomitant fetal growth disorders, need for iatrogenic preterm delivery and recurrent HDP. Adjustments for confounders (such as family history of cardiovascular diseases, high BMI, hypertension, diabetes, and dyslipidemia) do not eliminate, but attenuate this relationship. The underlying mechanisms have not been fully elucidated, but a concomitance of pre-pregnancy predisposition to increased risk of cardiovascular disease and a direct effect of pregnancy on the cardiovascular system may play a role in determining this excess of cardiovascular morbidity and mortality in women who experienced PE and GH in pregnancy. Guidelines regarding timing and extent of cardiovascular follow-up as well as strategies of prevention after HDP are lacking, but it is reasonable to recommend that screening should be started as early as 1 year after delivery and should primarily include awareness of the women of their future increased CV risk and lifestyle modifications with weight control, smoking cessation, healthy diet, and daily exercise. Future studies should address the issue of a structured screening for cardiovascular disease and the impact of timely preventive intervention in improving cardiovascular health in this group of young women.

## Author Contributions

AK, BT, and KM conceived the study. Articles were examined by AM, VG, AR, and KM. KM drafted the paper. All authors contributed in writing and revising the paper.

### Conflict of Interest

The authors declare that the research was conducted in the absence of any commercial or financial relationships that could be construed as a potential conflict of interest.
